# Macrophages in Renal Injury, Repair, Fibrosis Following Acute Kidney Injury and Targeted Therapy

**DOI:** 10.3389/fimmu.2022.934299

**Published:** 2022-07-13

**Authors:** Hui Chen, Na Liu, Shougang Zhuang

**Affiliations:** ^1^ Department of Nephrology, Shanghai East Hospital, Tongji University School of Medicine, Shanghai, China; ^2^ Department of Medicine, Rhode Island Hospital and Alpert Medical School, Brown University, Providence, RI, United States

**Keywords:** macrophages, kidney, inflammation, repair, regeneration, fibrosis, therapy

## Abstract

Acute kidney injury (AKI) is a renal disease with a high incidence and mortality. Currently, there are no targeted therapeutics for preventing and treating AKI. Macrophages, important players in mammalian immune response, are involved in the multiple pathological processes of AKI. They are dynamically activated and exhibit a diverse spectrum of functional phenotypes in the kidney after AKI. Targeting the mechanisms of macrophage activation significantly improves the outcomes of AKI in preclinical studies. In this review, we summarize the role of macrophages and the underlying mechanisms of macrophage activation during kidney injury, repair, regeneration, and fibrosis and provide strategies for macrophage-targeted therapies.

## Introduction

Acute kidney injury (AKI) has been defined as a clinical syndrome of kidney damage and acute loss of kidney function that induces acid-base and electrolyte disturbances, accumulation of nitrogenous waste products, and dysregulation of extracellular volume (1). Following mild AKI, the surviving tubular cells can undergo dedifferentiation, migration, and proliferation that result in morphological and functional recovery of the renal epithelium, but, under severe AKI, the kidney cannot be completely repaired, leading to permanent kidney damage and fibrosis. Depletion of macrophages can improve renal function and attenuate pathological changes following AKI in LysM-Cre mice (2, 3). However, the role of macrophages hyperactivation in AKI and AKI progression of fibrosis has not been well studied.

Although kidney tissue retains a certain number of resident macrophages under physiological conditions, circulating monocytes/macrophages are recruited to the renal interstitium when the kidney is injured (4). The macrophages are active and exhibit pro-inflammatory, regenerative, and resolving activities during the injury and repair processes. Macrophages have high plasticity and can change their phenotype in response to renal injury (5). Based on their activation states and functions, macrophages are divided into two types: classically activated macrophages (M1-type) and alternatively activated macrophages (M2-type) (6, 7). M1 macrophages are characterized by their ability to guide acute inflammatory responses, and can secrete multiple chemokines and pro-inflammatory cytokines, present antigens, and participate in positive immune responses (8–10). In contrast, M2 macrophages can help to resolve inflammation through high endocytic clearance capacities and the production of trophic factors that promote angiogenesis and mediate wound healing (11). However, M2 macrophages can also produce some profibrotic growth factors, such as epidermal growth factor (EGF) and transforming growth factor-β1 (TGF-β1) to induce the activation of interstitial fibroblasts and produce the intracellular matrix under severe or repeated renal injury. Damaged tubular cells release chemokines that promote M2 macrophage infiltration *via* selectin, integrin, trans-endothelial migration into the kidney and constantly produce growth factors/cytokine (12). Thus, M1 and M2 macrophages display different functions in the kidneys following AKI.

In this article, we review the role of macrophages and the underlying mechanisms of macrophage activation during kidney injury, repair, regeneration, and fibrosis and provide macrophage-targeted therapeutic strategies.

## Macrophages in Kidney Injury, Repair, Regeneration, and Fibrosis

### Macrophages Induce a Pro-Inflammatory Response Following AKI

Monocytes derived from bone marrow myeloid progenitors are acknowledged to be the main source of infiltrating macrophages in kidney disease (13). In experimental models and human biopsies, interstitial and glomerular macrophage infiltration can be detected in almost all types of AKI and chronic kidney disease (CKD) (14). The present understanding of macrophage functions in AKI derives mainly from studying various types of murine injury models, including nephrotoxin, ischemia reperfusion (IR), and rhabdomyolysis (15). Macrophages identify the primary damage signals through pattern recognition receptors (PRRs), a receptor of pathogen-associated molecular patterns (PAMPs), and recognize damage-associated molecular pattern (DAMPs) (16). NOD-like receptors (NLRs) and Toll-like receptors (TLRs) are the two main types of DAMPs. In AKI, there is TLR receptor-driven immunopathology, and resolution of inflammation for rapid regeneration of injured renal cells (17). A study conducted by Yin et al. (18) shows that inhibiting NLRP3 inflammasome activation in macrophages can significantly alleviated AKI. Activation of DAMPs/PAMPs through PRRs stimulates macrophage phagocytosis, antigen presentation, phagolysosome maturation, and the production of the pro-inflammatory cytokine TNF-α, reactive oxygen species (ROS), and IL-1β (19, 20). Overexpression of cytokine factors may induce severe kidney damage by boosting the inflammatory action (21) and promoting renal cell apoptosis and accelerating kidney injury (22). Necrotic muscle cells in rhabdomyolysis-induced AKI release heme-activated platelets that could raise the production of macrophage extracellular traps (METs) by enhancing histone citrullination and intracellular ROS generation (23). These findings emphasize the importance of understanding macrophage functions in human AKI pathophysiology.

### Macrophages in Renal Repair and Regeneration

It has been reported that the initial pro-inflammatory monocyte transition to macrophages stimulates tubular repair and regeneration from AKI (24). Currently, the functional changes of macrophages from pro-inflammatory to repair are still uncertain, however, this may be related to the anti-inflammatory effect of phagocytes and the changes in the microenvironment during kidney injury that cause transcriptional changes in resident macrophages (25). It has been demonstrated that macrophages contribute to the process of endogenous repair by secreting IL-22 and colony-stimulating factor (CSF)-1 cytokines (26). The recombinant IL-22 cytokine has the strongest pro-regenerator effects, as indicated by the observation that it promotes re-epithelialization after TEC injury (27). The TEC-specific CSF-1r/CSF-1 pathway also acts as a contributor to the repair processes (28). One study indicates that renal CSF-1 plays a vital role in polarizing renal macrophages to a M2 phenotype and mediating *in situ* proliferation and differentiation of renal epithelial cells during the recovery phase after kidney injury (29). Another study shows that granulocyte macrophage CSF (GM-CSF) mediates macrophage activation during the renal repair phase whereas the inhibition of GM-CSF attenuates the reparative macrophages and suppresses tubular proliferation after IR injury (30).

These are examples of how macrophages play a direct role in renal repair and regeneration. However, how macrophages promote renal repair and regeneration by interacting with other cell types is still incompletely understood.

### Macrophages in Renal Fibrosis

Macrophages have received great attention for their role in the progression of AKI to CKD in recent years. Although it has been reported that following AKI, the failure of macrophages to switch from pro-inflammatory M1 to the reparative M2 phenotype can stimulate progressive renal inflammation and fibrosis (31), sustained M2 macrophage infiltration of the kidney can produce profibrotic growth factors/cytokines, such as TGF-β1, resulting in excessive deposition of extracellular matrix (ECM) and the progression of renal fibrosis (12). Compelling evidence has confirmed that the degree of pro-inflammatory macrophage infiltration is significantly related to the severity of renal injury in CKD (32, 33). M1 macrophages are considered necessary for maintaining the pro-inflammatory condition, causing progressive renal injury and renal fibrotic development (34). Thus, both M1 and M2 types of macrophages are involved in the progression of renal fibrosis (35).

Renal fibrosis occurs when the ECM gradually replaces the normal renal tissue (36). It has been reported that eliminating macrophages can decrease renal fibrosis in most instances, supporting the profibrotic functions of macrophages in kidney diseases (37). Elevated levels of inflammatory cytokines, which can be secreted by macrophages, such as IL-6, IL-1β, monocyte chemoattractant protein-1 (MCP-1), TNF-α, and macrophage inflammatory protein-1β (MIP-1β), have been detected in fibrotic kidneys (38–40). These pro-inflammatory cytokines can attract other inflammatory cells from the blood to permeate the vascular, mesangial, and interstitial areas. In response to inflammation, the intrinsic cells secrete growth factors and nephrotoxic cytokines to drive fibroblasts to become myofibroblasts and proliferation in the kidney (41). Myofibroblasts will continue to produce more ECM components. Consequently, the imbalance of ECM synthesis and degradation promotes the deposition of ECM components in the kidney, thereby induing glomerular sclerosis and renal fibrosis (42). Studies have confirmed that renal fibrosis is the common pathological process during the progression of AKI to CKD (43).

## The Pathways of Macrophage Pro-Inflammation

Macrophages play roles in retaining an organism’s integrity by either repairing tissue under sterile inflammatory conditions or participating in pathogen elimination (44). Abnormally functioning of macrophages can induce various diseases (45). When activated, macrophages can engulf and kill pathogenic microorganisms and release pro-inflammatory cytokines to stimulate inflammatory responses and accelerate kidney fibrosis (46). The major signaling pathways of macrophage pro-inflammation in AKI include Notch, NF-κB, PI3K-AKT, JAK-STAT, and necroptosis pathway.

### Notch Signaling Pathway

The Notch signaling pathway plays a crucial role in embryonic development that is related to the regulation of cell differentiation, proliferation, and apoptosis through cell interactions (47). The Notch pathway has also been reported to influence inflammation and macrophage functions by regulating macrophage polarization through controlling gene expression (48). The Notch signaling pathway can be activated in kidney injury, and stimulate the expression of IL-1β, IL-18, TNF-α and ECM proteins such as collagen IV (Col IV) and fibronectin (FN) (49). Moreover, Notch signaling can control hypoxia‐induced epithelial-mesenchymal transformation (EMT) in the fibrotic kidney. Hypoxia activates the “epithelial” Notch pathway directly by up‐regulating the Notch intracellular domain (NIC) and its ligand expression levels, which results in the increased expression of collagen, fibronectin, and Snail and decreased expression of E‐cadherin (50). Downregulation of the Notch signaling pathway can facilitate the progression of monocyte differentiation into macrophages in the presence of GM-CSF and stimulate M1 and inhibit M2 polarization in macrophages (51).

### NF-κB Pathway

The NF-κB pathway acts as a vital signaling pathway in response to a multitude of environmental and intracellular stimuli and guides coordinated responses at the cellular level (52). NF-κB is an inducible transcription factor that regulates inflammation and an array of immune responses (53). It can be activated through either canonical or non-canonical pathways (54). The canonical NF-κB pathway activation is a rapid and transient response to a wide range of stimuli, such as NF-κB1 p50, p65 and c-REL, which are also referred to canonical NF-κB family members (55), while the non-canonical NF-κB pathway involves the activation of the p100/RelB heterodimer leading to the generation of p52/RelB and the prolonged activation of NF-κB target genes in response to a more limited set of stimuli, like TNF-α, IKKα, IKKβ, TRLs, NLRs (56–58). M1 macrophages accelerate inflammation or kidney damage *via* NF-κB and a combination of transcription factors (12). Moreover, NF-κB inhibition has been demonstrated to decrease inflammatory responses and fibrosis in multiple kidney disease models (59).

### JAK-STAT Pathway

The JAK-STAT (Janus kinase/signal transducer and activator of transcription) signaling pathway is closely related to the phenotypic activity of macrophages (60). It mediates cytokine liberation and leukocyte recruitment in the progression of AKI (61). The enhanced and prolonged activation of JAK/STAT signaling pathway stimulates M1 to secrete some cytokines/chemokines such as IL-1β, TNF-α, IL-6, IL-12 and IFN-γ, which exacerbate kidney injury (62). A study conducted by Zhang et al. (63) confirmed that activation of the JAK/STAT signaling pathway promotes kidney injury. Thus, the JAK/STAT pathway may be involved in the process of macrophage mediated inflammation.

### PI3K-AKT/mTOR Pathway

The phosphatidylinositol 3-kinase (PI3K)/protein kinase B (AKT)/mammalian target of the rapamycin (mTOR) pathway is activated in most real diseases (64, 65). This signaling pathway plays an important role in macrophage adhesion, migration, invasion, metabolism, proliferation, and survival (66–68). In the process of AKI, the PI3K/AKT/mTOR signaling pathway mediates multiple receptor signal transforms, including PAMP receptors, insulin receptors, adipokine receptors, and cytokine receptors (69), which may affect renal repair or damage. Activation of the PI3K/AKT/mTOR signaling pathway can induce macrophage activation to affect metabolic processes *via* high mobility group box-1 protein (HMGB1), TLR4, TNF-α, IL-6 and IκBα stimulators, thereby regulating macrophage activation and metabolism (70, 71), and can inhibit the autophagy process in AKI to alleviate kidney injury (72). It has been reported that targeting the PI3K/AKT/mTOR signaling pathway can limit AKI by limiting the activity of pro-inflammatory cytokines (73, 74). Moreover, studies have demonstrated that the PI3K/AKT/mTOR signaling pathway components have an isoform-specific and distinct status in inflammatory diseases and macrophage biology by controlling miRNAs, inflammatory cytokines, and functions, including autophagy, phagocytosis, and cell metabolism in AKI (72).

### Necroptosis Pathway

The necroptosis signaling pathway promotes inflammation by activating the NLRP3 inflammasome and starting the caspase-1 process of macrophages to release mature IL-1β (75, 76). RIPK3 and MLKL can both induce cell death, causing inflammation by secreting intracellular molecules (77, 78). RIPK3-caspase is crucial in the procession of macrophage differentiation (79), while how necroptosis mediated NLRP3 inflammasome activation, and mechanisms underly AKI to CKD progression remain unclear. As in tubular cells, TWEAK (TNF-like weak inducer of apoptosis) increased RIPK3 expression in macrophages however there is nothing known about RIPK1 (80). Macrophages conditional knockout mice also are involved in decreasing expression of MCP-1, IL-6, IL-1β like in renal tubular cells. Moreover, RIPK3 in macrophages promotes NF-*κ*B–dependent release of proinflammatory cytokines and activates the NF-*κ*B signaling pathway, in turn, promoting macrophage-tubular crosstalk that recruits inflammatory responses from tubular cells (80). Evidence has shown that necroptosis plays a crucial part in the early progression of multiple types of AKI (81, 82). A study by Chen et al. (83) found that RIPK3 or MLKL gene deletion improves macrophage infiltration, and NLRP3 inflammasome activation with a decrease in IL-1β maturation and caspase-1 activation. This eventually reduces interstitial fibrogenesis that occurs after renal IR injury. That explains why the necroptosis signaling pathway is contained in renal fibrosis-induced macrophages.

### Mitochondrial Injury in the Regulation of Macrophages

In addition to the above signaling pathways, mitochondrial injury has been confirmed to stimulate classical macrophage activation (84). Mitochondrial biosynthesis promotes the initiation and maintenance of macrophage activation as they associate with an increase in aerobic glycolysis and potential pathways that can generate adenosine triphosphate (ATP), which play a key position in the bioenergetic metabolism of all cellular compartments (85). A decrease in ATP production, an increase in ROS and HIF-1α production, the release of cytochrome c, and disruption of mitochondrial cristae are also observed, supporting a role for mitochondria in AKI (86, 87). Changes in mitochondrial dynamics also contribute to the decrease in mitochondrial energetics following AKI (88).

## Pathway of Macrophage Regulated Renal Fibrosis

### TGF-β Pathway

TGF-β is a major pro-fibrotic factor widely expressed in various types of renal cells in the body and plays a key role in the occurrence and development of renal fibrosis (89). The infiltration of M2 macrophages in injured kidneys is considered an important source of TGF-β (90). Kim et al. (91) observed that TGF-β secretion from M2 macrophages was significantly higher than that from M1 macrophages in AKI, suggesting the critical role of M2 macrophage-producing TGF-β in renal fibrosis. Chung et al. (92) also revealed that TGF-β released from M2 macrophages can enhance the EMT process. Therefore, the excessive or prolonged production of TGF-β from M2 macrophages may promote the deposition of collagen and other ECM components causing fibrosis in the kidney (93). The activation of the TGF-β pathway can active Smad3, NLRP3, and Caspase-1 stimulators which ultimately exacerbated the progression of AKI, and lead to renal fibrosis (94) (**Table 1**).

**Table 1:** The stimulators in the signaling pathways of macrophage pro-inflammation and macrophage regulated renal fibrosis.

Signaling pathway	Stimulator	Reference
Notch	IL-1β, IL-18, TNF-α Col IV, FN	(49)
NF-κB JAK-STAT	NF-κB1 p50, p65, c-REL TNF-α, IKKα, IKKβ, TRLs, NLRs IL-1β, TNF-α, IL-6, IL-12, IFN-γ	(55) (56–58) (62, 63)
PI3K-AKT-mTOR Necroptosis	HMGB1, TLR4, TNF-α, IκBα TLRs, IL-1β NLRP3 MCP-1, IL-6, TWEAK	(70, 71) (77) (80, 81) (80)
Mitochondiral injury	ATP mtROS, HIF-1α, Cytochrome C	(85) (85, 86)
TGF-β pathway	Smad3, NLRP3, Capsase-1 Collagen	(94) (93)

## Macrophage Targeted Therapy

i) Inhibition of macrophage infiltration. C-C motif chemokine 2 (CCL2) is an important monocyte chemoattractant that plays a crucial part in immune regulation (95). CCL2 mediates fibrosis by stimulating monocyte/macrophage infiltration (96). Pharmacological inhibition and genetic blockade of CCL2 or its receptor CCR2 are protective factors in renal inflammation and fibrotic models (97).

Interferon regulatory factor 4 (IRF4) is a transcription factor from the IRF factor family that exerts regulatory functions in the immune system (98). Sasaki et al. (99) reported that deletion of interferon regulatory factor 4 (IRF4) can prevent kidney fibrosis after ischemic injury by decreased macrophage recruitment and activation in mouse model. Similarly, Chen et al. (100) confirmed that IRF4 deficiency can reduce kidney inflammation and fibrosis after acute kidney injury by suppressing macrophage infiltration.

ii) Inhibition of macrophages polarization. It has been shown that small molecule inhibitors of c-Jun amino-terminal kinase (JNK) play protective roles in kidney injury in different forms of kidney disease by inhibiting renal inflammation, apoptosis, and fibrosis (101). JNK inhibitors (CC-401) can suppress M1 polarization and maintain effectiveness in renal IRI induced fibrotic models (102). An experiment regarding the JNK signaling pathway in renal I/R injury showed that CC-401 suppressed JNK signaling in kidney obstruction and obviously decreased renal fibrosis induced by collagen IV deposition and interstitial myofibroblast accumulation (103). These functions were attributed to the inhibition of profibrotic molecule TGF-b1 and connective tissue growth factor gene transcription.

The JAK-STAT signaling pathway is a multipole transduction system that inspects cellular responses to incoming signaling ligands (104). Blockade of the JAK/STAT pathway in a murine model demonstrated decreased renal interstitial fibrosis by inhibiting macrophage-induced inflammation (105). Meanwhile, Chuang et al. (106) indicated that inhibiting JAK improved renal function pathologic lesions and reduced macrophages accumulation in AKI.

Inhibition of necroptosis signaling pathway may also limit the progression of AKI to CKD (83). The NLRP3 inflammasome in macrophages is activated by various non-microbial danger signals released by necrotic cells and is considered an important pathway that sustains the inflammation-fibrosis cycle in CKD (107). NLRP3 inhibition using 1,3-butanediol treatment induces a shift of infiltrating renal macrophages from pro-inflammatory and pro-fibrotic to an anti-inflammatory phenotype and prevents renal fibrosis (108). A small-molecule NLRP3 inflammasome inhibitor, MCC950, can effectively reduce renal inflammation and fibrosis. Also, it may protect mouse renal function from different kinds of kidney damage. It is well established that hypertension is associated with the increased expression of adhesion molecules and pro-inflammatory cytokines and with the accumulation of inflammatory T cells and macrophages in the kidneys (109). Ismael et al. (110) found that MCC950 could restrain NLRP3 inflammasome activation of bone marrow-derived macrophages, monocyte-derived macrophages, and peripheral blood mononuclear cells in humans. Renal interstitial collagen deposition and the profibrotic cytokine TGF-β were blocked by treating with MCC950.

## Conclusions

Macrophages participate in the progression of AKI to CKD being involved in renal repair and regeneration. The multifunctional roles of macrophages depend on the microenvironment, including other cells of the immune system, cytokines, growth factors, and the macrophage-to-myofibroblast transition. Macrophages play an important role in diverse pathological processes such as renal inflammation and fibrosis. Accordingly, targeting the signaling mechanisms involved in macrophage recruitment and activation may alleviate renal functional deterioration in macrophage-dependent kidney diseases. In previous studies, the study of macrophages usually focused on their typing and polarization, especially macrophages recruited due to inflammation. Recent studies show that resident macrophages in the kidney are also critically involved in the process of kidney injury, repair, and fibrosis (111). The proliferation and activation of resident macrophages are closely related to renal injury and repair. In addition, epigenetic regulation has been reported to affect recruitment of macrophages and the release of inflammatory factors (112, 113). To date, only a few articles have described epigenetic mechanisms regulating macrophages in kidney diseases (112, 113). Therefore, elucidating epigenetic regulation in macrophage functions, phenotype changes and activation of local macrophages in the kidney following diverse injuries will be interesting future research directions.

## Data Availability Statement

The original contributions presented in the study are included in the article/supplementary material. Further inquiries can be directed to the corresponding author.

## Author Contributions

HC designed and wrote the manuscript; NL and SZ edited the manuscript. All authors contributed to the article and approved the submitted version.

## Funding

This work was supported by the US National Institutes of Health (2R01DK08506505A1to SZ), the National Natural Science Foundation of China (81830021 and 82070700 to SZ), and National key R&D Program of China (2018YFA0108802 to SZ).

## Conflict of Interest

The authors declare that the research was conducted in the absence of any commercial or financial relationships that could be construed as a potential conflict of interest.

## Publisher’s Note

All claims expressed in this article are solely those of the authors and do not necessarily represent those of their affiliated organizations, or those of the publisher, the editors and the reviewers. Any product that may be evaluated in this article, or claim that may be made by its manufacturer, is not guaranteed or endorsed by the publisher.
